# The effect of low-level red and near-infrared photobiomodulation on pain and function in tendinopathy: a systematic review and meta-analysis of randomized control trials

**DOI:** 10.1186/s13102-021-00306-z

**Published:** 2021-08-14

**Authors:** Nicholas Tripodi, Jack Feehan, Maja Husaric, Fotios Sidiroglou, Vasso Apostolopoulos

**Affiliations:** 1 Institute for Health and Sport, Victoria University, Room 1.16, 301 Flinders Lane, Melbourne, VIC 3000 Australia; 2 First Year College, Victoria University, Melbourne, Australia; 3 Australian Institute for Musculoskeletal Science (AIMSS), The University of Melbourne and Western Health, St. Albans, Australia; 4 Department of Medicine-Western Health, Melbourne Medical School, The University of Melbourne, St. Albans, Australia; 5 Institute for Sustainable Industries and Liveable Cities, Victoria University, Melbourne, Australia

**Keywords:** Tendinopathy, Photobiomodulation, Pain, Low-level laser therapy, Meta-analysis, Systematic review

## Abstract

**Background:**

Tendinopathy is a common clinical condition that can significantly affect a person’s physical function and quality of life. Despite exercise therapy being the mainstay of tendinopathy management, there are many potential adjunct therapies that remain under investigated, one of which is photobiomodulation (PBM). PBM uses varied wavelengths of light to create a biological effect. While PBM is used frequently in the management of tendinopathy, high quality evidence supporting its utility is lacking.

**Methods:**

A systematic search of the Pubmed, CINAHL, SCOPUS, Cochrane Database, Web of Science and SPORTSDICUS databases was performed for eligible articles in August 2020. Randomized Control Trials that used red or near-infrared PBM to treat tendinopathy disorders that made comparisons with a sham or ‘other’ intervention were included. Pain and function data were extracted from the included studies. The data were synthesized using a random effects model. The meta-analysis was performed using the mean difference (MD) and standardized mean difference (SMD) statistics.

**Results:**

A total of 17 trials were included (*n* = 835). When compared solely to other interventions PBM resulted in similar decreases in pain (MD -0.09; 95% CI − 0.79 to 0.61) and a smaller improvement in function (SMD -0.52; 95% CI − 0.81 to − 0.23). When PBM plus exercise was compared to sham treatment plus exercise, PBM demonstrated greater decreases in pain (MD 1.06; 95% CI 0.57 to 1.55) and improved function (MD 5.65; 95% CI 0.25 to 11.04). When PBM plus exercise was compared to other interventions plus exercise, no differences were noted in pain levels (MD 0.31; 95% CI − 0.07 to 0.70). Most studies were judged as low-risk of bias. The outcome measures were classified as very low to moderate evidence quality according to the Grading of Recommendation, Development and Evaluation tool.

**Conclusion:**

There is very-low-to-moderate quality evidence demonstrating that PBM has utility as a standalone and/or adjunctive therapy for tendinopathy disorders.

**Trial registration:**

PROPERO registration number: CRD42020202508.

**Supplementary Information:**

The online version contains supplementary material available at 10.1186/s13102-021-00306-z.

## Background

Tendinopathies represent a common presentation to clinical practice, particularly in active persons [1]. For instance, Achilles tendinopathy has been reported to occur at a rate of 2.35 per 1000 patients [2], whilst occurring between 6.2–9.5% in athletic populations [3]. Regardless of cohort, tendinopathy can profoundly affect a person’s quality of life and ability to perform activities of daily living, and cause considerable economic impact [4]. Traditionally, tendon pain was known as tendinitis, referring to the pain and inflammation thought to be associated with this condition [4]. However, as research in this area advanced, it was noted that most painful tendon disorders are chronic disorders, lacking a primary inflammatory driver [5–7]. Hence, the next term that evolved to describe this disorder was tendinosis, referring to the deleterious histopathological changes that can occur within a painful tendon [5]. More contemporary research now advocates for the term tendinopathy when describing any painful tendon disorder [7, 8]. Despite the original definition being grounded in the histopathological and clinical findings [7], tendinopathy is now defined as persistent tendon pain and loss of function related to mechanical loading [8], which may be associated with radiological changes [9].

Despite extensive research efforts in recent years, the complete pathophysiological picture of tendinopathy remains poorly understood [1]. However, it is known that four key cellular changes typify tendon pathology: 1. Increased number and metabolism of tenocytes; 2. Large proteoglycan presence, causing increased water content; 3. Abnormal collagen alignment and 4. New blood vessel and nerve growth within the tendon [10]. Regardless of the exact pathophysiological mechanisms, diagnosis of tendinopathy is primarily clinical, rather than radiological [1]. Tendinopathy presents as localized tendon pain that is correlated to mechanical load, that is beyond the tendon’s current capacity [8]. A clinician must pay close attention to changes in activity load and other rheumatological, metabolic and endocrine risk factors, with pain being produced during specific provocative movements, or by activities of daily living [1]. Furthermore, given the poor correlation between pain, function and histopathological radiological findings [10], and the absence of a defined nociceptive tendinopathic pathway [1], it is also important to consider the psychosocial influences of tendinopathy [1, 4, 11].

Due to the common prevalence of tendinopathy there is a large variety of treatment methodologies that have been employed, of which, exercise rehabilitation is the most well supported [1, 12, 13]. There are also a number of adjunct therapies used in the management of tendinopathy, including: Extracorporeal shock wave therapy (ESWT), Non-steroidal anti-inflammatory drugs (NSAIDs), injection therapies such as platelet rich plasma (PRP), corticosteroids (CS), and prolotherapy, transdermal application of CS through the method of Iontophoresis, and also passive interventions such as stretching and deep friction massage [1, 13]. While some of these treatments show promise, most have been shown to be no better, or worse that exercise rehabilitation [1].

An emerging and underexplored treatment in the management of tendinopathy is photobiomodulation (PBM) [14]. While the exact physiological mechanisms underpinning PBM are yet to be fully described, the prevailing theory is based on the interplay between adenosine triphosphate (ATP), nitric oxide (NO) and cytochrome c oxidase (complex IV of the mitochondria) [15]. It is thought that both red and near-infrared (NIR) light have a high affinity for CCO [15]. During routine metabolism, or in instances of cellular stress, NO may competitively bind to CCO, displacing oxygen, slowing or limiting ATP production. PBM has been suggested to displace the NO from CCO, allowing oxygen to more freely interact with CCO, thus enhancing ATP production [15]. Despite this mechanism being widely accepted, there is no evidence to date that shows a direct photo-biological interaction with CCO [14, 16]. Additionally, there are many other secondary mechanisms by which PBM may exert its effects. These include an increased production of reactive oxygen species (ROS), which can lead to upregulations in gene transcription and downstream protein expression [14, 17], and additionally may modulate key immune cells leading to improved tissue healing and neural fibre inhibition [14, 18, 19].

At a more fundamental level, how PBM affects tendon tissue in vitro, and in animal models has been investigated. In vitro PBM appears to influence multiple mechanisms related to growth and proliferation. Specifically, PBM can increase the expression of genes related to proliferating cell nuclear antigen (PCNA) and transforming growth factor-β1 (TGF-β1) [20, 21]; Cyclins E, A, and B1 [21]; expression of genes related to type I collagen, decorin [22] and dynamin II [23], all of which are key regulators of the healing response. Interestingly, PBM has also been shown to decrease the expression of genes related to inflammation such as TNF-α [24] and IL-6 in tenocytes [25]. The positive effects of PBM have also been observed in animal models of tendinopathy, showing mild improvements in functional healing compared to non-irradiated controls [26]. However, as with many areas of study within the field of PBM, a recent review article reported that the lack of a standardized process for treating animal tendons with PBM makes comparison difficult, and its further development and standardization should be given priority [27].

The impact of PBM on tendinopathy has been appraised with reviews on specific tendinopathies such as: lateral elbow tendinopathy [28]; Achilles tendinopathy [29]; and shoulder tendinopathy [30]; all of which demonstrated mixed effects, possibly due to a lack of consistent PBM application variables between studies. There has also been a systematic review and meta-analysis of the effects of PBM on all human tendinopathies, however it was reported in 2010, and included both randomized controlled trials (RCTs) and controlled clinical trials (CCTs) [31], and again mixed results were reported. Building on these previous works, and given the proposed universal effects of PBM, the aim of this work was to synthesize the current evidence describing the impact of low-intensity red and NIR PBM on pain and function in all tendinopathy disorders in human patients. Specifically, appraising only RCTs, we analyzed the effects of PBM on tendinopathy in three domains: Pain, PROMS and Strength.

## Methods

### Protocol and registration

This review was prospectively registered in the PROSPERO database (registration number: CRD42020202508). It was also completed and structured according to the Preferred Reporting Items for Systematic Reviews and Meta-Analyses (PRISMA) guidelines [32].

### Eligibility criteria

Studies included in this review were any randomized controlled trials that used up to a class 3B power laser, or equivalent light sources within the 600 nm – 1100 nm spectrum, to treat any diagnosed tendinopathy or tendinopathy-related disorders. Given the proposed universal effects of PBM, and the wide-ranging appraisal aim of this review, all tendinopathy and tendinopathy-related disorders were pooled. Comparisons had to be made to placebo or other clinical interventions in human adults. Further, the trials needed to report Visual Analogue Scale (VAS), validated Patient Reported Outcome Measure (PROM) data and/or changes in muscle strength. Studies were excluded if they were produced before the year 2000 given the change in both the diagnosis and understanding of tendinopathy [7] and the changes in PBM application [33] in that time. Articles unavailable in English were excluded.

### Information sources and search strategy

The search terms used in this review were: (Photobiomodulation OR Low-level laser OR LLLT) AND (tendon* OR tendin* OR epicond* OR teno* OR elbow OR bursitis OR subacromial). The databases that were searched were: Pubmed, CINAHL, SCOPUS, Cochrane Database, Web of Science, SPORTSDiscus. This search was completed by 1st August, 2020. An updated search was performed in April 2021 and yielded no additional results. Reference lists of relevant PBM reviews were also searched. A detailed description of the search can be found in Table 1 of Additional file 1.

### Study selection

The titles and abstract of all the studies yielded in the initial search were screened by two of the authors (NT and JF) for eligibility using the Covidence (Melbourne, Australia) platform. Any disagreements were resolved by a third author (MH). From here, full-text analysis was completed by the two of the authors (NT and JF) and again resolved by a third (MH). The authors of studies which reported insufficient data for the meta-analysis were contacted by email, however, were excluded if no response was given.

### Risk of Bias

Two of the authors (NT and JF) assessed the included studies for bias using the Cochrane Collaboration’s risk-of-bias tool [34]. Publication bias was assessed by funnel plot analysis generated by Review Manager Version 4.5 (The Cochrane Collaboration, Denmark), where there were more than 10 studies to analyze.

### Data collection process

Data of interest was extracted individually by two of the authors (NT and JF), with any disputes or inconsistencies resolved by the addition of a third author (MH), and then reaching a consensus decision.

### Data items

The primary outcomes taken for this study were pain intensity, in the form of the VAS, validated PROMS and changes in muscle strength. Range of motion measurements were excluded as they are not considered to be a core domain of tendinopathy [35]. The secondary outcome taken was reporting of adverse effects.

### Summary measures

As the primary measurements were all reported as continuous data, VAS and PROM data were combined using the mean difference (MD) statistic, while change in muscle strength data was analyzed using the standardized mean difference (SMD) statistic (given the heterogeneity in measuring muscle strength), using the change scores between time points. As only three of the included studies reported the SD change score [36–38], the correlation coefficient was calculated to be 0.8 based on these studies [39]. The data then underwent a sensitivity analysis comparing the meta-analysis results using a correlation coefficient of 0.2 and 0.8. As no change in the results were detected with either coefficient, the correlation coefficient of 0.8 was used for the final analysis VAS data was reported on a scale of 0–10, with data reported on a scale of 0–100 transformed to the 0–10 scale. PROM data was reported on a scale of 0–100. Studies that reported multiple VAS sub-scales (i.e. VAS rest, VAS night, etc.) and strength testing measurements means were averaged, and their standard deviation pooled according to previously described measures [39]. Studies that reported a 95% confidence interval (CI), and not the SD, were converted to SD [39].

### Synthesis of results

Two authors (NT and JF) completed the analysis using both Microsoft Excel (Microsoft, USA) and Review Manager Version 4.5 (The Cochrane Collaboration, Denmark). A random effects meta-analysis was used to analyze the results, with the I^2^ statistic being used to assess study heterogeneity. The trials were grouped according to VAS, specific PROM and strength measurements. Given the variability in design amongst the included studies, multiple subgroupings were made according to time points analyzed and comparison treatments and controls. ‘End of treatment’ was defined as end of a 2–4 week course of the treatment intervention, while ‘Follow Up’ was defined as 3 months post-treatment.

The evidence quality of each outcome was subjectively assessed using the Grading of Recommendation, Development and Evaluation (GRADE) tool [40]. Using the criteria from Tomazoni, Almeida [41], five factors and threshold criteria were used to assess the evidence quality: Risk of Bias: > 25% of trials classified at high risk of bias; Inconsistency: I^2^ > 50%; Indirectness: > 50% of participants not related to trial’s target audience; Imprecision: < 400 participants in the comparison for continuous outcomes; and Publication Bias: funnel plot if > 10 trials in same comparison [41]. The evidence quality could be categorized according to four ratings: High; Moderate; Low; and Very Low. Each time an outcome did not meet each of the threshold criteria it was downgraded one level per criteria. For example, if one measure did not meet the thresholds for risk of bias and Inconsistency it was classified as low-quality evidence, downgraded from high-quality evidence.

## Results

### Search summary

**Fig. 1:**
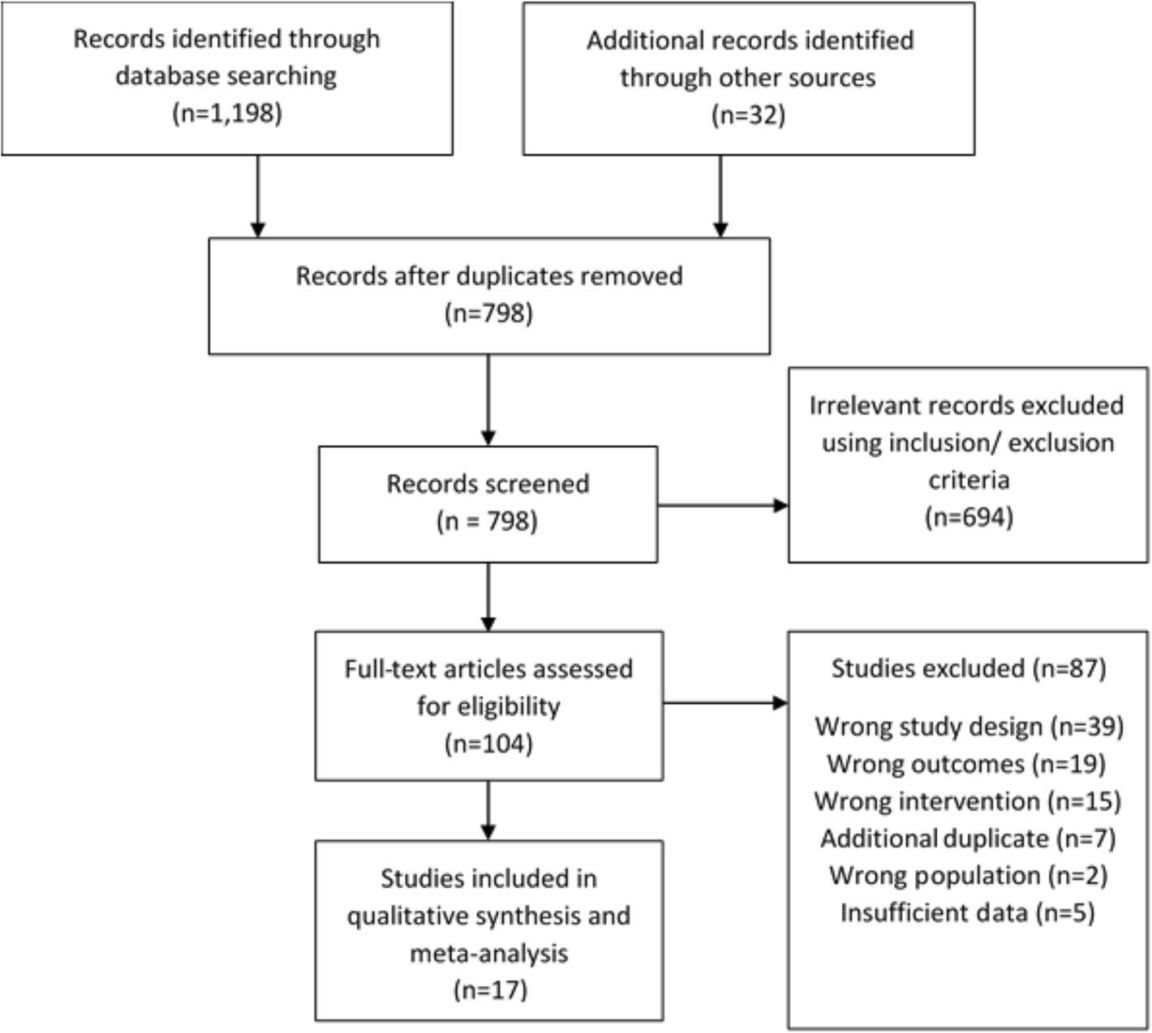
Literature search process according to the PRISMA guidelines

The detailed search strategy is shown in Table 1 of Additional file 1. The initial search strategy yielded 1230 results, after title and abstract screening of these results, 104 studies remained. When these were subjected to full-text screening 22 studies were eligible, of which 17 were included in the meta-analysis [36–38, 42–55] (Fig. 1). The five eligible, but excluded studies, were omitted due to insufficient data, which could not be obtained by contacting the authors [56–60]/ The pooled studies equated to a total of (*n* = 835) participants.

### Included study characteristics

#### Participant diagnosis

Of the included studies, one investigated (*n* = 1) Achilles Tendinopathy (AT) [53]; one investigated De Quervain’s Tenosynovitis (DQT) (*n* = 1) [51]; seven (*n* = 7) investigated Lateral Elbow Tendinopathy (LET) [36, 43, 45, 46, 48, 50, 52]; one (*n* = 1) investigated Patella Tendinopathy (PT) [38]; and seven (*n* = 7) investigated Sub-acromial Syndrome/Rotator Cuff Tendinopathy (SAS/RT) [37, 42, 44, 47, 49, 54, 55] (Table 1).

**Table 1:** Characteristics of included studies

Study First Author, Year	Diagnosis	Total Participants; Participants per group	Intervention Groups	Outcomes Extracted	Treatment Time	Measurement Time Points
Abrisham 2011 [42]	SAS	80; 40/40	PBM + Exercise, Sham; Laser + Exercise	VAS	Two weeks	1. Baseline; 2. Two weeks
Baktir 2018 [43]	LET	37; 12/13/13	PBM; Phonophoresis; Iontophoresis	VAS; PRTEE-t	Three weeks	1. Baseline; 2. Two Weeks
Bal 2009 [44]	SAS	44; 22/22	PBM + Exercise; Exercise Only	VAS; SPADI-t	Two weeks	1. Baseline; 2. Two weeks; 3. Three month follow up
Celik 2019 [45]	LET	43; 23/22	PBM + Exercise; ESWT + Exercise	VAS; DASH	Four weeks	1. Baseline; 2. Four weeks; 3. Three month follow up
Devrimsel 2014 [46]	LET	60; 30/30	PBM; ESWT	VAS	Four weeks	1. Baseline; 2. Four weeks; 3. Three month follow up
Dogan 2010 [47]	SAS	52; 30/22	PBM + Exercise; Sham PBM + Exercise	VAS; SAPDI-t	Three weeks	1. Baseline; 2. Three weeks
Emanet 2010 [36]	LET	50; 25/25	PBM + Exercise; Sham PBM + Exercise	VAS; DASH; PRETEE-t	Three weeks	1. Baseline; 2. Three weeks; 3. Three month follow up
Eslamian 2012 [37]	RT	50; 25/25	PBM + Passive Physiotherapy; Sham PBM + Passive Physiotherapy	VAS; SDQ	Three weeks	1. Baseline; 2. Four weeks; 3. Three month follow up
Kaydok 2020 [48]	LET	59; 30/29	PBM + HILT	VAS; QDASH	Three weeks	1. Baseline; 2. Three weeks
Kibar 2017 [49]	SAS	62; 30/32	PBM; Sham PBM	VAS; SAPDI-t	Three weeks	1. Baseline; 2. Three weeks
Lam 2007 [50]	LET	39; 21/18	PBM + Exercise; Sham + Exercise Only	VAS; DASH	Three weeks	1. Baseline; 2. Three weeks
Liu 2014 [38]	PT	21; 7/7/7	PBM; Exercise Only; PBM + Exercise	VAS; VISA-P	Four Weeks	1. Baseline; 2. Four weeks
Sharma 2015 [51]	DQT	30; 15/15	PBM; US	VAS	Two Weeks	1. Baseline; 2. Two weeks
Stergioulas 2007 [52]	LET	50; 20/20	PBM + Exercise; Sham + Exercise	VAS	Four and Eight Weeks	1. Baseline; 2. Eight weeks; 3. Two month follow up
Stergioulas 2008 [53]	AT	40; 20/20	PBM + Exercise; Sham + Exercise	VAS	Four and Eight Weeks	1. Baseline; 2. Four weeks; 3. Eight Weeks; 4. Three month follow up
Yavuz 2014 [54]	SAS	31; 16/15	PBM + Exercise; US + Exercise	VAS; SPADI-D	Four Weeks	1. Baseline; 2. Four weeks; 3. Three month follow up
Yeldan, 2009 [55]	SAS	60; 34/26	PBM + Exercise; Sham PBM + Exercise	VAS; DASH; SDQ	Three Weeks	1. Baseline; 2. Three weeks

#### Interventions

There were a wide array of PBM application variables used within the included studies. All the studies used NIR light, ranging from 0.5-5 J/cm^2^, and all studies irradiated multiple sites. Additionally, there were a number of studies that did not report all necessary light application variables [36, 42, 46, 47, 49, 51, 54, 55] (Tables 1 and 2). Other comparative interventions (“other interventions”) included: Phonophoresis and Iontophoresis [43]; ESWT [46]; High-Intensity Laser Therapy (HILT) [48]; Passive Physiotherapy [37]; and US [51]; with the remaining studies using exercise alone [36, 42, 50, 52, 53, 55], or exercise plus another intervention [45, 54]. Only four studies used the WALT guidelines [33] to inform their treatment protocols [36, 51, 53, 54] (Tables 1 and 2).

**Table 2:** PBM variables of included studies

Study First Author, Year	PBM light source; Wavelength	Light source power output during treatment (mW)	Fluence per spot (J/cm^**2**^)	Treatment spots	PBM sessions per week; Total PBM sessions	WALT recommendations informed trial?
Abrisham 2011 [42]	‘Laser Device;’ 890 nm	Not Reported	2–4	3	5; 10	No
Baktir 2018 [43]	GaAs Laser Diode; 904 nm	0.12	Not Reported	5	5; 15	No
Bal 2009 [44]	GaAs Laser Diode; 904 nm	13.2	2	4	5;10	No
Celik 2019 [45]	GaAs Laser Diode; 904 nm	40	2.4	6	3;12	No
Devrimsel 2014 [46]	‘Laser;’ 850 nm	Not Reported	Not Reported	Not Reported	2; 10	No
Dogan 2010 [47]	GaAlAs; 850 nm	Not Reported	5	5–6	4–5; 14	No
Emanet 2010 [36]	GaAs Laser; 905 nm	Not Reported	1	2	5; 15	Yes
Eslamian 2012 [37]	Ga-Al-As Laser Diode; 850 nm	100	4	Up to 10	3; 9	No
Kaydok 2020 [48]	Ga-Al-As Laser Diode; 904 nm	240	2–4	6	3; 9	No
Kibar 2017 [49]	Ga-Al-As Laser Diode; 850 nm	Not Reported	4	11	3; 9	No
Lam 2007 [50]	Ga-Al-As Laser Diode; 904 nm	25	2.4	Average 2.4	3; 9	No
Liu 2014 [38]	Ga-Al-As Laser Diode; 810 nm	200	Not Reported	3	6; 24	No
Sharma 2015 [51]	Ga-Al-As Laser Diode; 830 nm	30–40	3	Not Reported	3–4; 7	Yes
Stergioulas 2007 [52]	Ga-As; 904 nm	40	2.4	6	1–2; 12	No
Stergioulas 2008 [53]	Ga-Al-As Laser Diode; 820 nm	30	0.5	6	1–2; 12	Yes
Yavuz 2014 [54]	Ga-Al-As Laser Diode; 850 nm	Not Reported	3	5 maximum	2–3; 10	Yes
Yeldan, 2009 [55]	GaAs; 904 nm	Not Reported	Not Reported	5 Maximum	Not Reported	No

#### Outcome measures

All the included studies used VAS as an outcome measure. Of the studies that used PROMS in their measures, four (*k* = 4) studies used the Disabilities of the Arm, Shoulder and Hand (DASH) measure [36, 45, 50, 55]; with one (*k* = 1) using the Quick DASH (Q-DASH) [48]; two (*k* = 2) used the Patient Reported Tennis Elbow Evaluation (PRTEE) [36, 43]; two (*n* = 2) used the Shoulder Disability Questionnaire (SDQ) [37, 55]; three (*k* = 3) used the Shoulder Pain and Disability Index (SPADI) [44, 47, 49]; and one (*k* = 1) study used the Victoria Institute of Sport Assessment-Patella Tendon (VISA-P) [38]. Due to the heterogeneous nature, and limited numbers of study interventions, only the DASH scores could be subject to meta-analysis. Additionally, there were 10 (*k* = 10) studies that used muscle strength scores and an outcome measure [36, 38, 43, 45, 46, 48, 50–52, 55] (Table 1). Only five studies reported if any adverse effects occurred in the trial, of which there were none [42, 44, 47, 48, 55].

### Risk of Bias

**Fig. 2:**
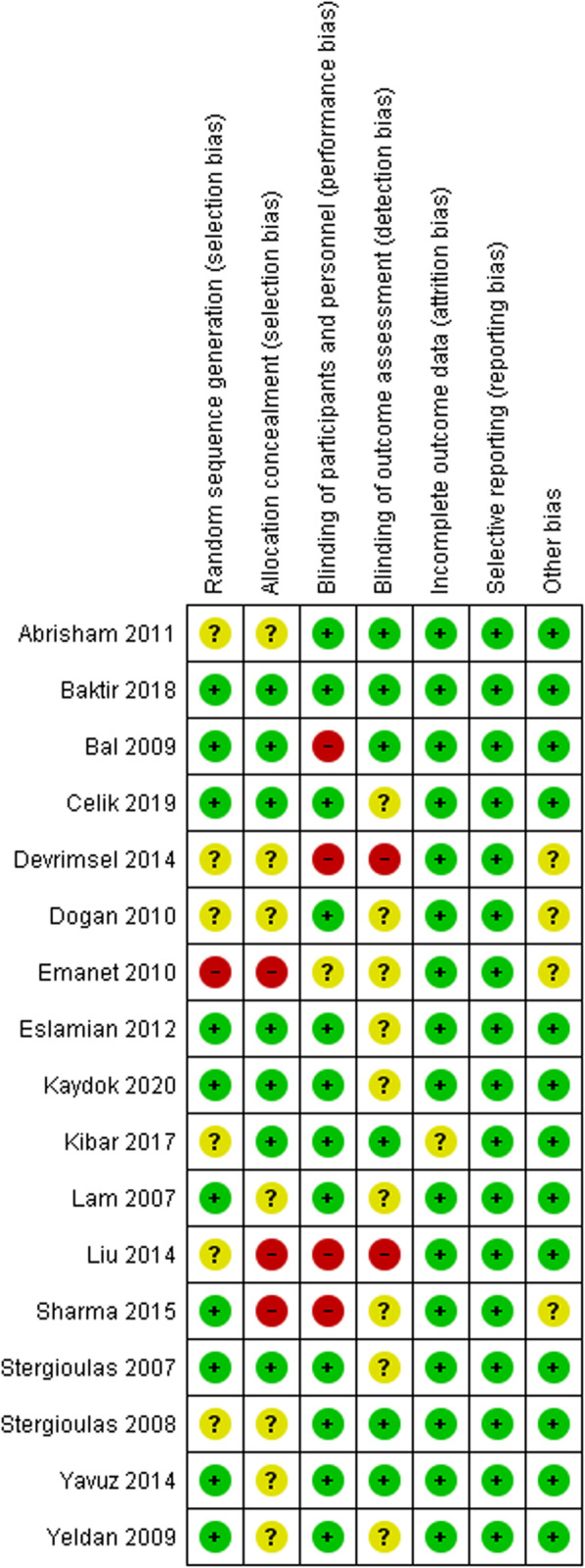
Risk of bias summary - review authors’ judgements about each risk of bias item for each included study

When pooled together the included studies were judged to a low risk of bias 68.1% of the time, an unclear risk of bias 23.5% of the time, and high risk of bias 8.4% of the time. Largely, the included studies tended to under report the randomization and blinding protocols, with some studies also failing to report all the required light parameters, hence being judged as being subject to ‘other bias’ (Fig. 2). Publication bias via funnel plot analysis was not completed as none of the individual forest plots contained > 10 studies [34].

### VAS measures

#### PBM only versus other interventions only

**Fig. 3:**
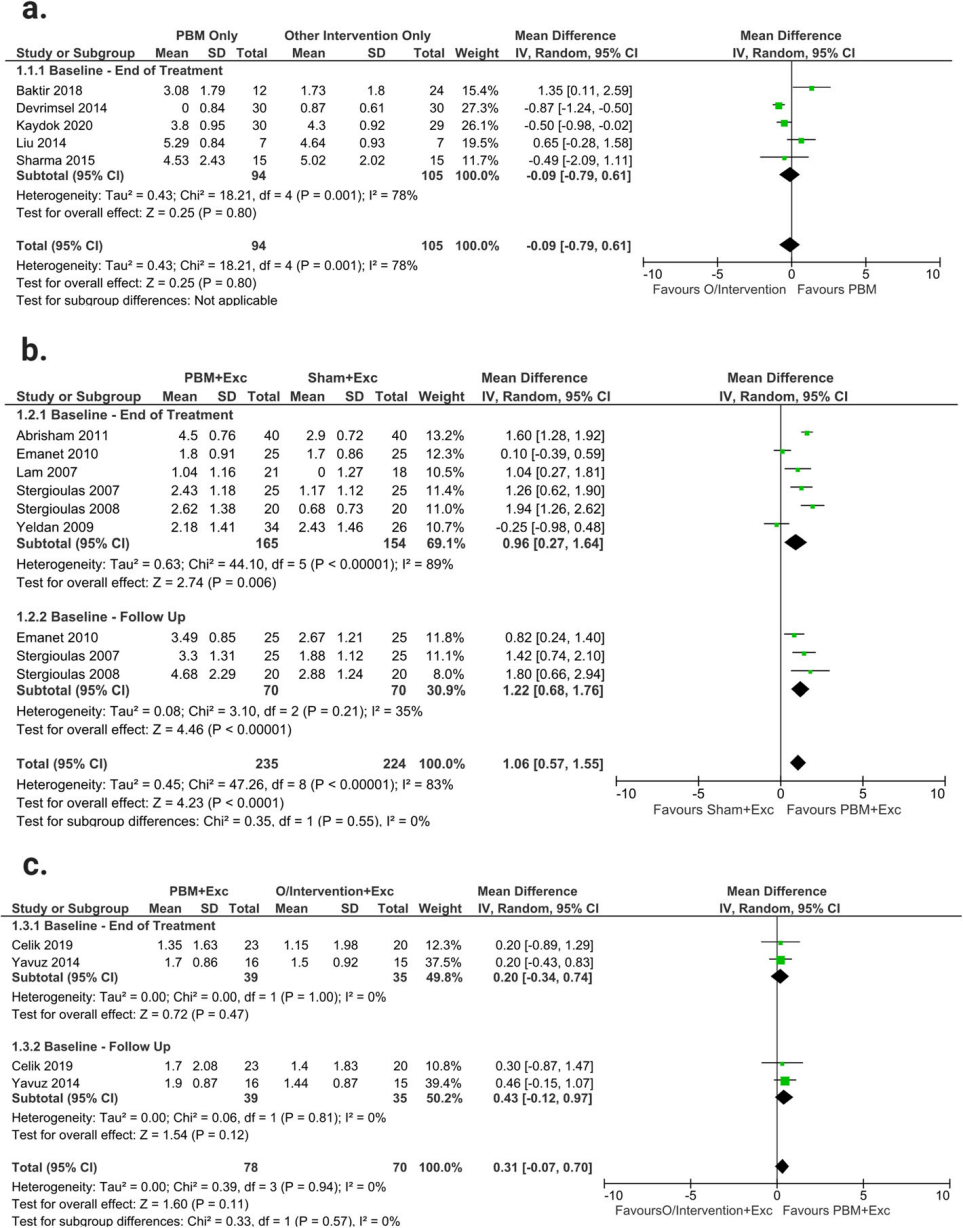
VAS: **a**: Forest plot of comparing PBM only and other interventions (O/Intervention) only; **b**: Forest plot of the effects of PBM plus exercise (Exc) versus sham treatment plus exercise; **c**: Forest plot of the effects of PBM plus exercise versus other interventions plus exercise

When compared to other interventions only (Phonophoresis, Iontophoresis, ESWT, HILT, CS Injection and US), PBM only, demonstrated similar effects from baseline-end of treatment (MD -0.09; 95% CI --0.79 to 0.61; I^2^ = 78%; *n* = 105). The studies in this outcome were downgraded to very low-quality evidence due to risk of bias, inconsistency, and imprecision (Fig. 3a).

#### PBM plus exercise versus sham plus exercise

Overall, PBM plus exercise demonstrated significant reductions in pain levels compared to sham plus exercise (MD 1.06; 95% CI 0.57 to 1.55; I^2^ = 82%; *n* = 224). The time period subgroup analysis showed similar results with, PBM plus exercise creating a more substantial decrease in pain at baseline-end of treatment (MD 0.96; 95% CI 0.27 to 1.64; I^2^ = 89%; *n* = 154), and baseline-follow up (MD 1.22; 95% CI 0.68 to 1.76; I^2^ = 35%; *n* = 70). There were no significant between-subgroup differences found (*p* = 0.55). The studies in this outcome were downgraded to low-quality evidence due to inconsistency and Imprecision (Fig. 3b).

#### PBM plus exercise versus other intervention plus exercise

No significant difference was found between PBM plus exercise and other interventions (ESWT and US) plus exercise (MD 0.31; 95% CI − 0.07 to 0.70; I^2^ = 0%; *n* = 70). The time period subgroup analysis demonstrated similar effects on pain within the baseline-end of treatment (MD 0.20; 95% CI − 0.34 to 0.74; I^2^ = 0%; *n* = 35), and baseline-follow up (MD 0.43; 95% CI − 0.12 to 0.97; I^2^ = 0%; *n* = 35) periods. There were no significant between-subgroup differences found (*p* = 0.57). The studies in this outcome were downgraded to moderate-quality evidence due to imprecision (Fig. 3c).

### Proms

#### DASH: PBM plus exercise versus sham plus exercise

**Fig. 4:**
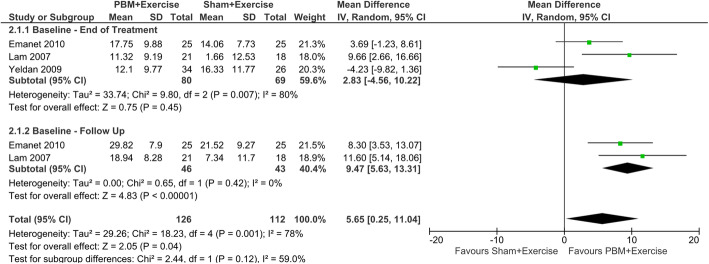
PROMS: Forest plot of comparing PBM plus exercise versus sham + exercise

PBM plus exercise demonstrated a significant improvement in the DASH PROM score compared to sham plus exercise (MD 5.65; 95% CI 0.25 to 11.04; I^2^ = 78% *n* = 112). The time period subgroup analysis showed no significant effect of PBM at baseline-end of treatment (MD 2.83; 95% CI − 4.56 to 0.70; I^2^ = 80%; *n* = 69), while PBM plus exercise demonstrated a significant positive effect at the baseline-follow up period (MD 9.47; 95% CI 5.63 to 13.31; I^2^ = 0%; *n* = 43). There were no significant between-subgroup differences found (*p* = 0.12). The studies in this outcome were downgraded to very low-quality evidence due to risk of bias, inconsistency and imprecision (Fig. 4).

### Strength measures

#### PBM only versus other interventions only

**Fig. 5:**
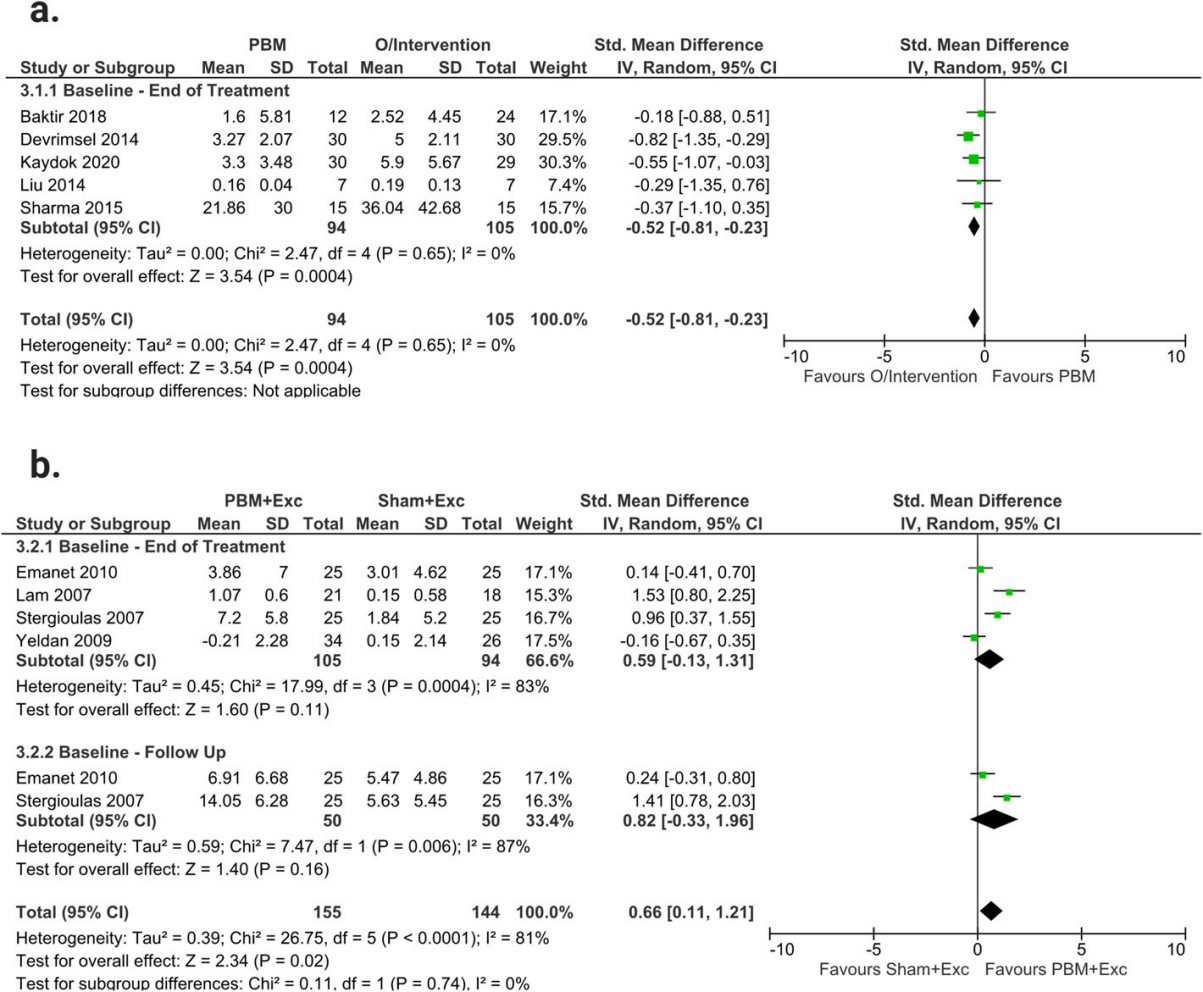
Strength Measures: **a**: Forest plot of comparing PBM only and other interventions (O/Intervention) only; **b**: Forest plot of the effects of PBM plus exercise (Exc) versus sham treatment plus exercise

When compared to other interventions only (Phonophoresis, Iontophoresis, ESWT, HILT, CS Injection and US), PBM only, demonstrated a significantly decreased effect from baseline-end of treatment (SMD -0.52; 95% CI − 0.81 to − 0.23; I^2^ = 0%; *n* = 105) (Fig. 5a). The studies in this outcome were downgraded to low-quality evidence due to risk of bias and imprecision.

PBM plus Exercise versus Sham plus Exercise.

Overall, the results demonstrated that PBM plus exercise caused significant increase in strength compared to sham plus exercise (SMD 0.66; 95% CI 0.11 to 1.21; I^2^ = 81%; *n* = 144). The time period subgroup analysis however, demonstrated no significant effect for PBM plus exercise on functional strength measures within both the baseline-end of treatment (SMD 0.59; 95% CI − 0.13 to − 1.31; I^2^ = 83%; *n* = 94) and baseline-follow up period (SMD 0.82; 95% CI − 0.33 to 1.96; I^2^ = 87%; *n* = 50). There were no significant between-subgroup differences found (*p* = 0.74). The studies in this outcome were downgraded to low-quality evidence due to Inconsistency and Imprecision (Fig. 5b).

### GRADE classifications

The quality of evidence classification for each outcome is located in Table 2 in Additional file 1.

## Discussion

The overarching aim of this review was to investigate the effect of low-intensity red and NIR PBM on pain and function in patients with tendinopathy and tendinopathy-related disorders. It was found that when compared to other interventions, with or without exercise added (Phonophoresis, Iontophoresis, ESWT, HILT, CS Injection and US), that there is very low-moderate quality evidence to show that PBM with or without exercise were equally effective at reducing pain. This review also found very low-quality evidence demonstrating that when PBM is combined with exercise, it results in a significant improvement in PROMS compared to sham treatment plus exercise. There was also low-quality evidence demonstrating that other interventions (Phonophoresis, Iontophoresis, ESWT, HILT, CS Injection and US) were significantly better at improving functional strength measures compared to PBM, while when exercise was added to PBM therapy, it was significantly better at restoring functional muscle strength compared to sham treatment plus exercise.

Despite the small body of somewhat favorable evidence for PBM, as a whole, there were multiple limitations with the studies included in this review. Firstly, according to the GRADE classification system, all outcome measure assessed were classified as very low, low, or moderate quality of evidence. This was largely due to many of studies been classified as inconsistent (I^2^ > 50%) and imprecise (< 400 participants per outcome measure) and judged to be at high risk of bias (> 25% trials are classified as high risk). Although the imprecision could be addressed with the inclusion of more studies, the fact that we were not able to assess for publication bias, as no outcomes had more the 10 included trials, is something that will have to be addressed in future trials and reviews. Furthermore, 31.9% of the risk of bias variables assessed were judged to be of unknown or high-risk of bias, which should be taken into account when interpreting the results of this review.

It is well documented throughout the literature that the inconsistent nature of PBM experiments, both clinical [41, 61] and in vitro [14], are a significant hurdle in establishing both a concrete physiological mechanism, and a widely used and accepted set of clinical implementation guidelines. Appraising the studies included in this review, we see many differing forms of PBM application, including total number of treatments, treatment sites, and irradiation per site. This is understandable given they are treating different areas of tendon pathology, however, there were some studies that did not report all the required treatment variables [36, 42, 46, 47, 49, 51, 54, 55], making exact replication challenging, in the process affecting the quality of evidence. The WALT (World Association for Laser Therapy) recommendations are a set of therapeutic recommendations for clinical and scientific application of red and NIR spectrum PBM [33]. Only four of the trials in this review referenced the WALT recommendations in their study design [36, 51, 53, 54], further underlining the need for higher levels of inter-study consistency.

Heavy strength and plyometric training, in addition to training load management, appear to be the most efficacious exercise modalities to employ during tendinopathy management [1]. This review demonstrated very low-quality evidence that PBM could be used as an adjunct therapy to enhance the effects of exercise rehabilitation. That said, a limitation of this analysis was that all the exercise modalities from each study were pooled in each outcome measure, hence different exercise prescriptions may have affected the results. Future research in this area should more stringently control the exercise prescription groups in line with tendinopathy best practice. Interestingly, this review also found that when compared to other interventions, PBM was equally as effective at decreasing pain, however, this was again limited by the pooling of all other interventions. Many of the other interventions that used a pharmacological anti-inflammatory agent, such as Phonophoresis, Iontophoresis and CS Injection, can cause unwanted patient side effects [62]. In fact, it is now recommended that practitioners move away from these methods, CS injections in particular, due to the long-term deleterious tissue effects they can have [62]. In light of this, PBM may represent a non-invasive, cost effective and safe alternative to the more traditional injection and anti-inflammatory based therapies used in tendinopathy management. However, more robust trials are needed to elucidate this effect.

To our knowledge only one other systematic review and meta-analysis has been performed on the effect of PBM on all tendinopathies previously [31]. This review demonstrated similar mixed results concerning the effects of PBM on pain and function in tendinopathy and similar issues with evidence quality to the present review, despite having fewer studies available for analysis. Tendinopathy specific systematic review and meta-analyses have been conducted for shoulder [30] and Achilles tendinopathy [29] and similarly to this review, found a mixed efficacy of PBM underpinned by trials of moderate-very low evidence. Taking these findings together, it is clear that more widespread and robust RCTs are needed to better inform the use of PBM in tendinopathy management.

The strengths of this review include a detailed search of multiple databases, as well as additional searches of paper reference lists. Further, two of the authors performed the entire search process and the risk of bias and GRADE categorization, with a third author resolving any disputes. Another limitation of this study was the fact that all tendinopathies were pooled together as a single diagnostic entity. Hence, the analysis may not have accounted for the heterogeneity of tendinopathy disorders. However, the analysis appeared to indicate similar effects of PBM, regardless of specific diagnosis. More specific-tendinopathy RCTs are needed to underpin more robust single-tendinopathy systematic reviews and meta-analyses. Additionally, the exclusion of multiple studies whose required statistics were unobtainable from either the paper, or the contact authors may have changed the study results. As previously stated, the future research focus of PBM for the management of tendinopathy should be set on performing repeated robust RCTs that adequately report and justify all treatment parameters and follow the Consolidated Standard of Reporting Trials (CONSORT) guidelines. This will firstly better elucidate if PBM is an effective standalone and/or adjunct therapy for PBM, and secondly if high-quality evidence is found for this effect, it will underpin improved treatment guidelines, potentially translating to improved patient health outcomes.

## Conclusion

PBM is an increasingly used treatment modality for a range of musculoskeletal disorders, however, there are many questions regarding its mechanisms and true effectiveness that remain under-investigated and unanswered. Currently, there is very-low-to-moderate quality evidence that low-intensity red and NIR PBM is an effective standalone and exercise-adjunctive treatment for tendinopathy disorders in humans. Further, a similar quality of evidence demonstrates that it may have utility as a less-invasive and more risk-averse adjunctive treatment to more traditional passive interventions. More robust RCTs that adhere to the CONSORT guidelines need to be performed to further elucidate its effectiveness.

## Supplementary Information

**Additional file 1: Table 1.** Review Search Strategy and Results. **Table 2.** GRADE Classifications.

## Data Availability

The Pubmed, CINAHL, SCOPUS, Cochrane Database, Web of Science and SPORTSDICUS databases were searched for eligible articles in August 2020′. Additionally, this study was registered with the PROSPERO database (registration number: CRD42020202508). All data and analysis can be made available on request.

## Abbreviations

SAS: Subacromial syndrome
LET: Lateral elbow tendinopathy
RT: Rotator cuff tendinopathy
PT: Patella tendinopathy
AT: Achilles tendinopathy
PBM: Photobiomodulation
ESWT: Extracorporeal shock wave therapy
HILT: High-intensity laser therapy
US: Ultrasound
VAS: Visual analogue scale
DASH: Disabilities of the arm, shoulder and hand measure
QDASH: Quick DASH
PRTEE: Patient reported tennis elbow evaluation
SDQ: Shoulder disability questionnaire
SPADI: Shoulder pain and disability index
VISA-P: Victoria institute of sport assessment-patella tendon
WALT: World association for laser therapy
Exc: Exercise
O/Intervention: Other Intervention
MD: Mean Difference
SMD: Standardized mean difference
CI: Confidence Interval
mW: Milliwatt
J: Joules
NIR: Near-infrared light
RCTs: Randomized controlled trials (RCTs)
CCTs: Controlled clinical trials
ATP: Adenosine Triphosphate
NO: Nitric Oxide
CCO: Cytochrome C Oxidase
PROMS: Patient reported outcome measures
PCNA: Proliferating cell nuclear antigen
ROS: Reactive oxygen species
