# Supplementary material for: The effect of low-level red and near-infrared photobiomodulation on pain and function in tendinopathy: a systematic review and meta-analysis of randomized control trials
Source: BMC Sports Sci Med Rehabil. 2021 Aug 14;13:91. doi: 10.1186/s13102-021-00306-z (PMC8364035; doi:10.1186/s13102-021-00306-z)
Supplement: Supplementary file 1 — Additional file 1: Table 1. Review Search Strategy and Results. Table 2. GRADE Classifications. [file 13102_2021_306_MOESM1_ESM.docx]

**E-APPENDIX: TITLE PAGE**

The effect of low-level red and near-infrared photobiomodulation on pain and function in tendinopathy: A systematic review and meta-analysis of randomized control trials

**Nicholas Tripodi^1,2,3*^ (ORCID: 0000-0001-5062-0409), Jack Feehan^1,3,4^ (ORCID: 0000-0002-9627-1299), Maja Husaric^1, 2^ (ORCID:** **0000-0001-5627-7541), Fotios Sidiroglou^2, 5^ ORCID: 0000-0002-9399-8484), Vasso Apostolopoulos^1^ (ORCID:** **0000-0001-6788-2771)**

^1^Institute for Health and Sport, Victoria University, Melbourne, Australia

^2^First Year College, Victoria University, Melbourne, Australia

^3^Australian Institute for Musculoskeletal Science (AIMSS), The University of Melbourne and Western Health, St. Albans, Australia

^4^Department of Medicine-Western Health, Melbourne Medical School, The University of Melbourne, St. Albans, Australia

^5^Institute for Sustainable Industries and Liveable Cities, Victoria University, Melbourne, Australia

**^*^** Corresponding Author:

Nicholas Tripodi
[nicholas.Tripodi@vu.edu.au](mailto:nicholas.Tripodi@vu.edu.au)
Victoria University, Room 1.16, 301 Flinders Lane, Melbourne, VIC 3000, Australia.

**E-Appendix, Table 1: Review Search Strategy and Results**

| **Database** | **Search Strategy** | **Number of Results** |
| --- | --- | --- |
| Pubmed | (Photobiomodulation OR Low-level laser OR LLLT) AND (tendon* OR tendin* OR epicond* OR teno* OR elbow OR bursitis OR subacromial) | 203 |
| CINAHL | (Photobiomodulation OR Low-level laser OR LLLT) AND (tendon* OR tendin* OR epicond* OR teno* OR elbow OR bursitis OR subacromial) | 97 |
| SCOPUS | (Photobiomodulation OR Low-level laser OR LLLT) AND (tendon* OR tendin* OR epicond* OR teno* OR elbow OR bursitis OR subacromial) | 482 |
| Cochrane Database | (Photobiomodulation OR Low-level laser OR LLLT) AND (tendon* OR tendin* OR epicond* OR teno* OR elbow OR bursitis OR subacromial) | 5 |
| Web of Science | (Photobiomodulation OR Low-level laser OR LLLT) AND (tendon* OR tendin* OR epicond* OR teno* OR elbow OR bursitis OR subacromial) | 353 |
| SPORTSDiscus | (Photobiomodulation OR Low-level laser OR LLLT) AND (tendon* OR tendin* OR epicond* OR teno* OR elbow OR bursitis OR subacromial) | 58 |
| Other Sources: Searching relevant PBM review references lists | N/A | 32 |
| **Total** | | **1,230** |

**E-Appendix, Table 2: GRADE Classifications**

*Grades Criteria*

- Risk of Bias : Yes if >25% trials are classified as high risk
- Inconsistency: Yes if I^2^ >50%
- Indirectness Yes if >50% of participants not related to trial's target audience
- Imprecision: Yes if <400 participants in the comparison for continuous outcomes
- Publication Bias: Yes if funnel plot if >10 trials in same comparison

*Overall Quality Criteria*

- High: 0 Yes responses
- Moderate 1 Yes response
- Low: 2 Yes responses
- Very Low: 3 or more Yes responses

| **Outcome** | **Risk of Bias** | **Inconsistency** | **Indirectness** | **Imprecision** | **Publication Bias** | **Overall Quality** |
| --- | --- | --- | --- | --- | --- | --- |
| VAS: PBM vs. Other Intervention | Yes | Yes | No | Yes | No | Very Low |
| VAS: PBM + Exercise vs. Sham + Exercise | No | Yes | No | Yes | No | Low |
| VAS: PBM + Exercise vs. Other Intervention + Exercise | No | No | No | Yes | No | Moderate |
| PROM – DASH: PBM + Exercise vs. Sham + Exercise | Yes | Yes | No | Yes | No | Very Low |
| Strength: PBM vs. Other Intervention | Yes | Yes | No | No | No | Low |
| Strength: PBM + Exercise vs. Sham + Exercise | No | Yes | No | Yes | No | Low |
